# Ethical meat: respect for farm animals

**DOI:** 10.1093/af/vfz052

**Published:** 2020-01-10

**Authors:** Giuseppe Pulina

**Affiliations:** Department of Agraria, University of Sassari, Sassari, Italy

**Keywords:** animal ethics, animal rights, humans’ obligations, meat

**Implications**

- Ethics is a human science and is not given in nature.
- Even if animals are sentient beings, this condition is not enough to possess a moral state.
- Animals are not bearers of rights but subjects of our precise duties.
- Food availability is a prevailing human interest, so meat consumption is morally justified.

The writings, positions, depth of the theme, ideal and, unfortunately, ideological contrasts on ethics and animals, are so abundant as to render any attempt to circumscribe the field and to draw judgments of merit anyway incomplete. Before anything else, ethics pursues the aim of improving the lives of men and other living beings while respecting the fundamental natural and cultural principles that govern the biosphere and human societies. This article chooses an operative position by discussing what good or evil means for animals and how this should be interpreted for positive purposes, including the production of food for humans.

Ethics is a human science and is not given in nature. Good and evil are cultural constructs, different in different social contexts, which evolve over time and which require solid dialectical bases to be affirmed and not slip into fallacious reasoning. To affirm therefore that a specific behavior is good as natural, is not an ethical reasoning, so to say that a natural behavior is good in itself, does not make sense.

Many examples could be given for this: for all it is enough to mention the unnecessary pain caused in animals which, if generated by Man, is considered reprehensible, if caused by other animals (carnivores, conspecifics, or competitors) is judged natural. From this premise, it follows that ethics is an exclusively human affair and that the moral behaviors deriving from it must be oriented by rational thought.

To circumscribe the field of discourse, this article tries to answer the question: is it right to raise and sacrifice animals to eat the meat, assuming that animals have rights and if so, which rights or if not, what are our obligations towards animals? (The recent Italian book by Andrea Bertaglio “In defense of meat” (2018) addresses in a simple and rigorous way, many aspects of the debate between supporters and detractors of the consumption of meat. With the quiet tones used in the various arguments, Bertaglio gives us a vademecum of “good education in dialogue,” in a World of which there is an increasing need.)

To begin to answer the question, we broadly follow the reasoning of the philosopher Hsiao (2015). (An exhaustive treatment of these themes can be found in the Italian book edited by Bertoni (2017).) Animals are sentient beings as different evidences have shown that it is possible to attribute their declarative knowledge without adopting an anthropomorphic perspective (Veisser et al., 2012). (The 2007 Lisbon Treaty in force since 2009, in art. 13, states: “In formulating and implementing the Union’s agriculture, fisheries, transport, internal market, research and technological development and space policies, the Union and the Member States shall, since animals are sentient beings, pay full regard to the welfare requirements of animals, while respecting the legislative or administrative provisions and customs of the Member States relating in particular to religious rites, cultural traditions and regional heritage.”) Experimental data from research in animal psychology push to consider that in different species, there are subjective states of which animals are conscious (Rassu et al., 2005; Le Neindre et al., 2017). Animal psychologists are dealing with the concept of personality, defined as “consistent individual differences in behaviour,” in which genetic effects are likened to irreversible developmental plasticity (Wilson et al., 2019). In a nutshell, animals experience suffering, so they are aware of it and try, as much as possible, to avoid it, but they have subjective emotions and have the ability to feel and pursue pleasure. However, being sentient, even with individual diversities, is not enough of a condition to possess a moral state. Causing pain in an animal is wrong, but it is not morally wrong; a good reason is not necessarily a moral reason because, to be such, it needs to refer to a moral fact. Animals do not possess a moral state as they are conscious and sentient beings, but they are not rational beings. Rationality is the presupposition for belonging to a moral community, since only rational beings can act morally. Moral actions are free actions that require the agent to have the ability to know the reason for the action and evaluate the consequences: knowledge and action are two essential objects of ethics. Then, morality is an informal and public system that applies to all rational individuals to govern any behavior that can have consequences on others, in order to avoid evil and pain. Therefore, although it is widely recognized that other nonhuman animals are in various degrees intelligent, they are not rational agents at all and are therefore not belonging to the moral community that is formed exclusively by man. Well-being is an interest of both humans and animals, but the interest of the first is moral; from this it follows that it is prevalent over that of the latter. Eating is a priority for human well-being, so it is a moral interest. Saving foods from animal pests, including mice, is morally justified. Similarly, eating the meat of animals is a priority for the well-being of man, so the moral interest of the latter prevails over the nonmoral interest of the former. The important thing is that humans do not cause animals unecessary suffering during breeding and in sacrifice.

Beyond the moral justification, is it right to suppress a life for food use, even in conditions according to some of nonindispensability? (Many supports the thesis that one can live even without feeding on products of animal origin. I recently wrote a book in Italian (Pulina, 2019) which demonstrates the opposite. Part of this article takes up a chapter of the book.) This is known as “The meat paradox” (people like to eat meat but do not like that animals have to suffer and be killed), first formulated by Loughnan et al. in (2012). In a more recent review, Ursin (2016) goes through three strategies to solve or neutralize the psychological tension that emerges in meat eaters due to the meat paradox: “first, the tension can be dealt with by changing one’s behavior to fit one’s values; second, it can be relieved by adjusting the meaning of one’s values to fit one’s behaviour; third, it is possible to uphold one’s values and adjust one’s perception of the phenomena to align one’s values with one’s behaviour.”

This philosopher concludes that the dilemma can be solved by either not eating meat or not worrying about killing animals, but he thinks that the virtuous omnivore should not be trapped between these two alternatives and can “accept the resulting tension as a sign of the complexity of our relations with animals.”

A study conducted in Australia indirectly provides us with the answer to the paradox. Archer (2011) states that to cultivate 1 hectare of grain, 55 sentient lives (mice and other small mammals or marsupials) are killed with great suffering compared with the 2.2 of cattle per hectare destined for a quick sacrifice. And this is also valid in the case in which the grains are used for fattening cattle or sheep, as most of the live weight is obtained from the grass of the pastures. In short, to speak of good or evil in killing an animal is less trivial than one might think, as Leroy and Praet (2017) show in a recent study describing the trajectory of the social perception of this act, from prehistoric hunting to modern slaughtering techniques used in the developed countries: deference, ritualization, professionalization, and removal from the collective consciousness (Figures 1–3).

**Figure 1.**
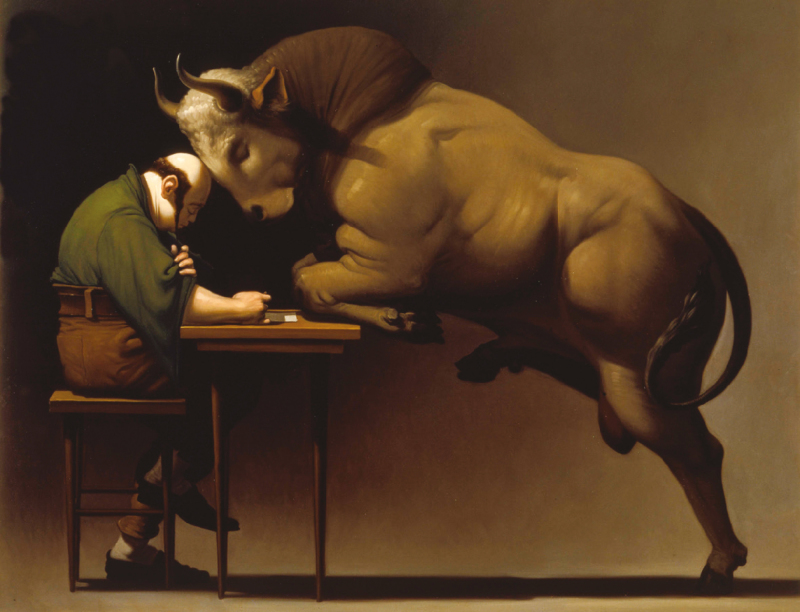
Wainer Vaccari (Modena 1949), Melancholia, 1992. Oil on canvas, 200 × 250 cm (private collection).

**Figure 2.**
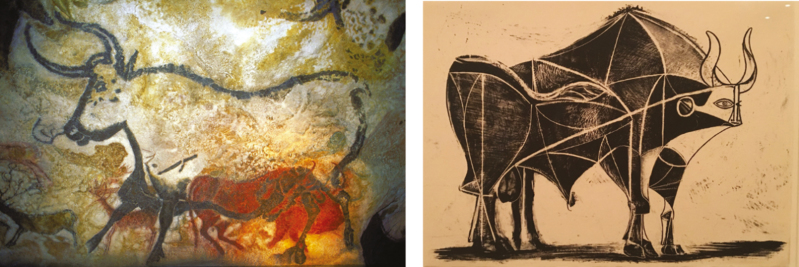
Rock painting of Lascaux (France), 15,000 years BC, left; Pablo Picasso 1946, El toro, 11 estado, n. 5. Picasso Museum, Malaga.

**Figure 3.**
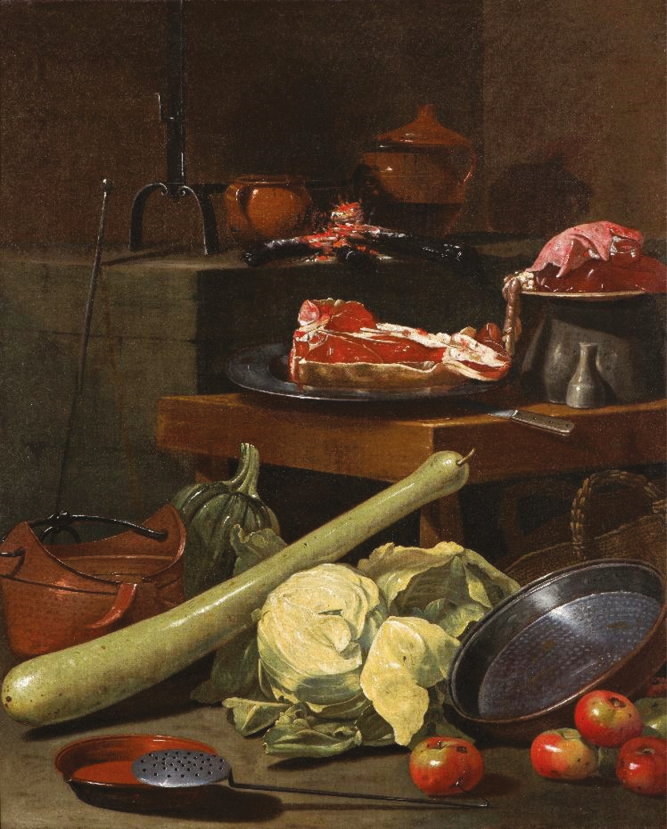
Cristoforo Munari (Reggio Emilia 1667–1720), Still life painting with Fiorentina steak. Oil on canvas, 147 × 117 cm (private collection).

On the other hand, taking on ethical issues aimed at the beginnings of life, birth and death, is very difficult when it comes to human affairs, and becomes even more complex when animals are involved. As a concrete example, the table of inference shown in Figure 4 is an example of the complexity of our moral position about humans and animals killing.

**Figure 4.**
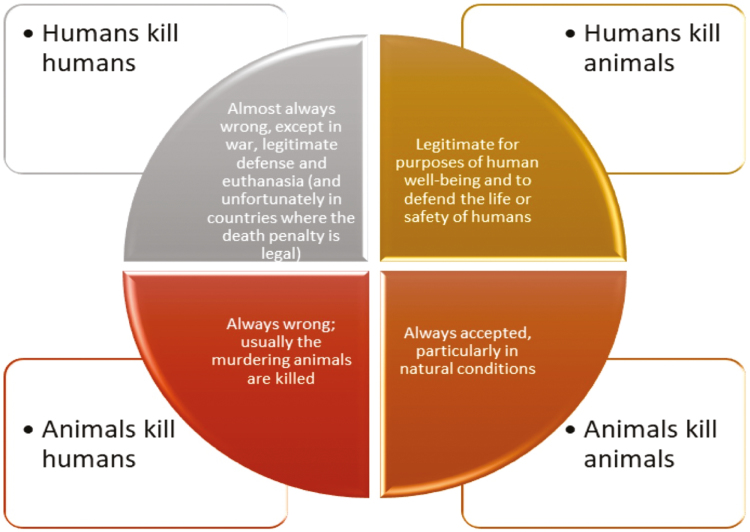
The complexity of the moral position about killing. There are some emerging issues related to artificial intelligence (AI) regarding the dilemma of an automated mechanism who must choose among different scenarios, in each of which there will be one or more humans injured or killed; or even the legitimacy of turning off an AI if signs of awareness emerge in the machine.

Another way to dissolve the paradox is to legitimize the rights of animals, including the right to life. According to Gozzano (2004), who takes up some of the concepts explained above, moral values evoked by human rationality are objective. These values are those that no one would reject based on the assumption that some rights should be formulated: species, intelligence, and recipients of interests. Animals should not be recognized owners of moral rights, but forms of respect must be applied to them, starting with the guarantee of well-being as sentient beings. It is noteworthy to note that “modern animal ethics embrace not only our duties and obligations to animals, but also our duties and obligations to animal users and society in general” (Mattews and Hemsworth, 2012). (Mattews and Hemsworth (2012) observe that “while attitudes to animals at the individual level influence how people behave towards animals, attitudes to animals at the community level can also influence the development of animal-related policy and legislation.”)

According to constitutional lawyer Gemma (2004), the best arrangement is the one that sees man enjoying a preferential position in the “social contract.” Protection of other species, although necessary, cannot go so far as to compromise the rights inherent in the human person (the so-called partial equality). However, in 1978, UNESCO adopted the Universal Declaration of Animal Rights, to be understood in this context as passive rights, or obligations of Man towards animals. Article 3, useful for our discussion on meat, states: “Animals must not be subjected to bad treatments or to cruel acts. If it is necessary to kill an animal, it must be instantaneous, painless and cause no apprehension. A dead animal must be treated with decency.”

Although most constitutionalists agree with Gemma (2004) in believing that animals do not hold constitutional rights, there are a growing number of countries with constitutions, though still few for now, which provide for the protection of nonhuman animals: Switzerland (as of 1973), India (1976), Brazil (1988), Slovenia (1991), Germany (2002), Luxembourg (2007), Austria (2013), and Egypt (2014). An extensive review on animal protection through constitutional charters at the global level can be found in World Animal Net (2014): “some nations reference the treatment of animals, specifically. Others reference such terms as fauna, species, living things, and nature. The level of protection and recognition afforded to animals through constitutions varies widely by country. Some constitutions, for example, have many provisions where others may contain only a single provision. Some require that all animals are deserving of protection, while others are concerned with rare or native species only. Similarly, the constitutions of many countries emphasize the importance of protection of species as national resources and assets, while others view animals as deserving of protection in their own right. Finally, some constitutions authorize the regulation of specific industries such as fishing, hunting, and slaughter.”

In a recent article (2018), Jessica Eisen explored the “tension between constitutional animal protection and prevailing theories of constitutionalism and proposes a supplementary account of constitutional theory that embraces the state’s obligation to attend to the interests of its most vulnerable members—even, and perhaps especially, where those members are incapable of constitutional self-assertion.”

Having established that animals are not bearers of rights, but subjects of our precise duties, the final question is what these duties are and how should we implement them. At the beginning of the 1960s, the recognition of conscious and sentient states of mind for animals led to the formulation by the Farm Animal Welfare Council of the five freedoms (the concept of Five Freedoms originated from the report of the Technical Committee to Enquire into the Welfare of Animals kept under Intensive Livestock Husbandry Systems, known as the Brambell Report (1965). The document stated that livestock animals should have the freedom to “stand up, lie down, turn around, clean and stretch their limbs,” a list that is sometimes still called Brambell’s Five Freedoms) (see Figure 5). This formulation has been adapted to farm animals. (Among the many, I quote the wording on the Ruminantia website, whose editor-in-chief is veterinary surgeon Alessandro Fantini (https://www.ruminantia.it/).)

1. Freedom from thirst, hunger, and bad nutrition. To comply with this first point, the needs relating to the quality, quantity, and frequency of meals given to the animals must be assessed, respecting the physiology, age, climatic conditions, etc.
2. Freedom to have an adequate physical environment. Recalls the importance of the right to live in an environment that is welcoming, that protects and that is adequate, that is not a source of discomfort for the animal. Slippery floors, or those that make cleaning difficult or still without insulation or shelters, are obviously not suitable for maintaining this condition.
3. Freedom from pain, wounds, and diseases. The state of illness causes a condition of discomfort that can be accentuated by inappropriate conditions of detention; in turn the disease may require special conditions and precautions for the maintenance of the animals. Here too, medical knowledge plays a primary role in preventing and interrupting suffering. The keepers must also be educated in the responsibilities related to the protection of the state of health and to recognize the manifestations of discomfort and pain. Ignoring or underestimating the pain perceived by the animal can determine survival risks, and failure to diagnose diseases can spread infectious diseases with the obvious consequences.
4. Freedom to exhibit normal species-specific behavioral characteristics. This point summarizes the five freedoms. In fact, when the animal “is not well,” it manifests a series of behaviors that move away from the norm.
5. Freedom from fear and discomfort. Animals have the right to be protected from events and stimuli that cause negative emotions: repeated fear and stress are incompatible with health. Those who have animals must be able to understand what the stressful events or stimuli are, their consequences on health and behavior and how to prevent them. Animal welfare is protected by law in several countries (Vapnek and Chapman, 2011), with specific rules concerning transport and slaughter. Those who do not respect them violate the law and must be rightly prosecuted. Just as all forms of animal abuse must be cut off: let us remember that, in most of the countries, this is a crime and is prosecuted by law.

**Figure 5.**
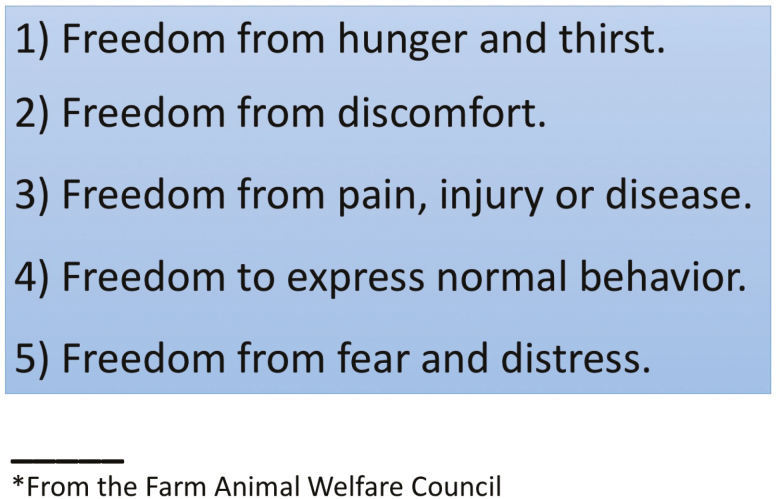
The five freedoms.

In conclusion, meat consumption is morally justified; animals are not carriers of rights; we have specific obligations towards them, first respecting them and guaranteeing their well-being. (A complete and open access scientific review on animal welfare is reported in the special issue of *Italian Journal of Animal Science* (Bertoni, 2009). A special issue of *Animal Frontiers* in the same topic, edited by Lay Donald C. Jr., was published in 2012.) However, since ethics is an operative science whose affirmations are influenced by the state of philosophical and scientific research, as well as by the evolution of common sentiment, the prevalence of antispeciesism in Western societies and its spreading in other cultures may, in a short time, make it necessary to reconsider the roles of animals in modern societies and to include them, with greater or lesser limitations, in the field of subjects with moral interests, and therefore actors of recognized rights.

## About the Author

**Giuseppe Pulina**, Ph.D. is Full Professor of Animal Science at the Department of AGRARIA, University of Sassari (Italy) where he teaches Ethics and sustainability of animal production for graduate students, and Ethics of scientific research for Ph.D. students. He was President of the National Conference of AGRARIA (former conference of the Deans of Agriculture) and, for a 3-year period (2010–2013) Vice President of AISSA (Association of Scientific Societies of Agriculture) and President of Association for Science and Animal Production (ASPA) of which he is currently President Emeritus. He is a member of the Accademia dei Georgofili of Florence, honorary member of the Pesaro-Urbino Academy of Agriculture, and a corresponding member of the Agricultural Academy of Bologna. He is coordinator of Assalzoo Scientific Committee and President of Carni Sostenbili (Sustainable Meat) Association.
